# Yaws Circulating in Nonhuman Primates, Uganda and Rwanda

**DOI:** 10.3201/eid3104.241562

**Published:** 2025-04

**Authors:** Tony L. Goldberg, Leah A. Owens, Julius Nziza, Richard Muvunyi, Jessica M. Rothman, Patrick Omeja, Colin A. Chapman

**Affiliations:** University of Wisconsin–Madison, Madison, Wisconsin, USA (T.L. Goldberg, L.A. Owens); Gorilla Doctors, Musanze, Rwanda (J. Nziza); Rwanda Development Board, Kigali, Rwanda (R. Muvunyi); Hunter College of City University of New York, New York, New York, USA (J.M. Rothman); New York Consortium in Evolutionary Primatology, New York (J.M. Rothman); Makerere University, Kampala, Uganda (P. Omeja); Vancouver Island University, Nanaimo, British Columbia, Canada (C.A. Chapman)

**Keywords:** yaws, bacteria, *Treponema pallidum pertenue*, nonhuman primates, zoonoses, East Africa, Uganda, Rwanda

## Abstract

The bacterium *Treponema pallidum pertenue* causes yaws in humans and nonhuman primates. We describe 33% *T. pallidum pertenue* seropositivity in 9 species of nonhuman primates in Uganda and Rwanda, seroconversion during a lethal outbreak and a novel bacterial genomic lineage. Yaws may threaten both public health and conservation in the region.

Yaws is a bacterial disease endemic to the tropics caused by *Treponema pallidum pertenue*, which is distinct from its conspecifics *T. p. endemicum*, the cause of bejel, and *T. p. pallidum*, the cause of syphilis (*1*,*2*). Yaws causes skin papules, crusts, and ulcers progressing to systemic infection and disfiguring skeletal disease (*1*). Yaws has been targeted for eradication by 2030, but challenges persist (*3*).

Eradicating yaws may be complicated by *T. p. pertenue* in wild nonhuman primates (*4*). Several monkey species across Africa have tested positive for serum antibodies to *T. p. pertenue* (*5*). Genetic analyses show phylogenetic interspersion of human and primate variants, implying historic host switching and, therefore, a potential reservoir role for primates (*6*–*8*). However, geographic ranges of hosts and of infection in African primates remain incompletely known, as does the extent of bacterial diversity in primates. Furthermore, few data exist on *T. p. pertenue* transmission within wild primates.

## The Study

**Figure 1:**
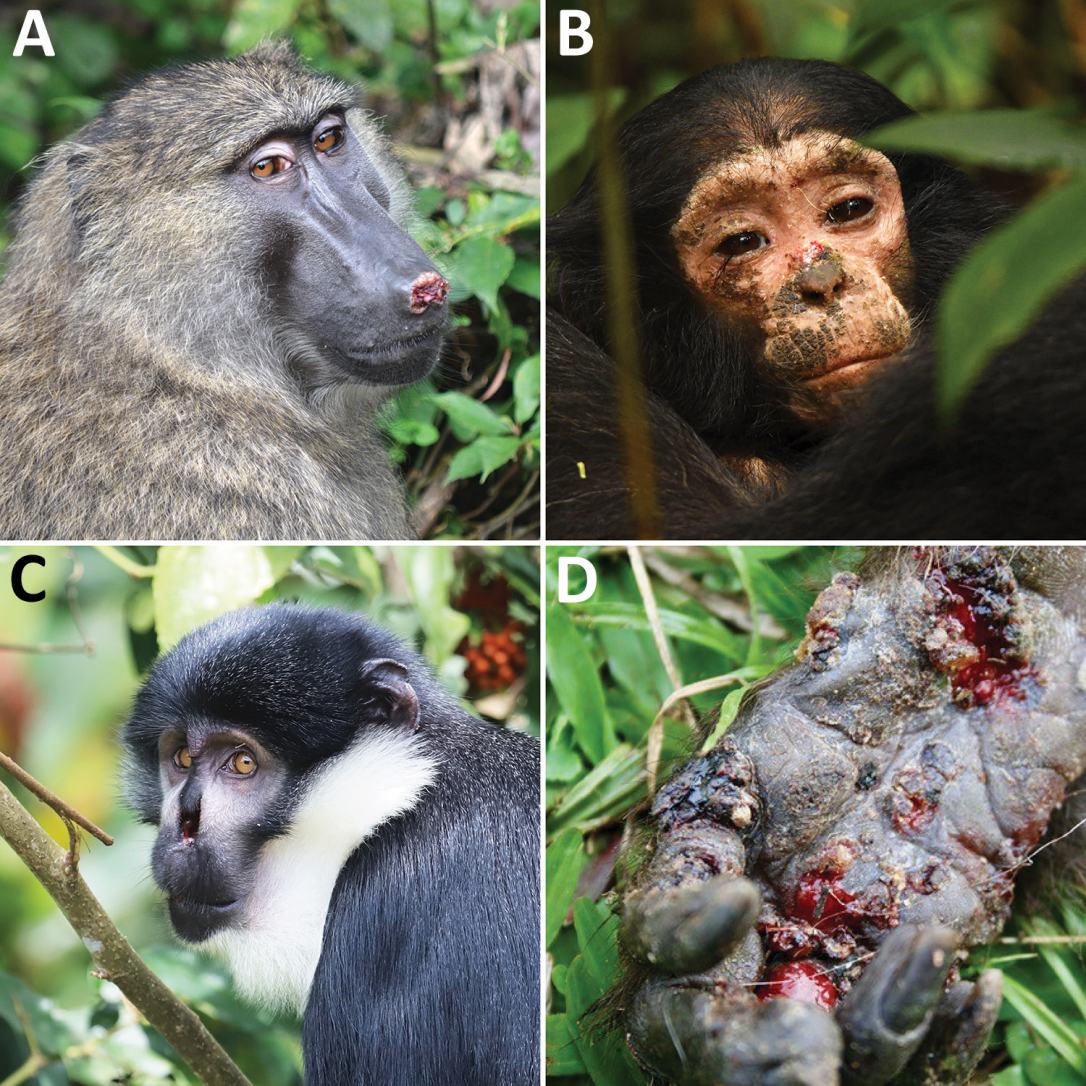
Primates in Kibale National Park, Uganda, showing clinical signs of yaws in study of yaws circulating in nonhuman primates, Uganda and Rwanda. A) Adult olive baboon (*Papio anubis*) with erosive lesion on nose. Photograph by Jessica Rothman. B) Juvenile eastern chimpanzee (*Pan troglodytes schweinfurthii*) with papules and crusting on face. Photograph by Kevin Lee. C) Adult L’Hoest’s monkey (*Allochrocebus lhoesti*) with eroded/missing nose. Photograph by Nancy Stevens. D) Adult red colobus monkey (*Piliocolobus tephrosceles*) with ulcerative lesions on hand. Photograph by Alicia Rich.

We screened 103 serum samples collected during 2005–2014 from apparently healthy primates in Uganda and Rwanda (Appendix Figure 1) using a commercial serologic test validated for primates (Appendix). Overall, seroprevalence was 33.0%, with 32.8% prevalence in Uganda and 33.3% in Rwanda (Table 1). Prevalence did not differ significantly between male (33.8%) and female (31.6%) primates (p = 1.000) but was higher in adults (41.3%) than in younger primates (14.3%) (p = 0.0105 by Fisher exact test). Prevalence range was 0%–76.9% among host species (χ^2^= 27.1; d.f. 8; 2-tailed p = 0.0007). Seroprevalence was particularly high in olive baboons (*Papio anubis*; 76.9%) and vervet monkeys (*Chlorocebus pygerythrus*; 26.7%), which are common in the region and frequently live alongside humans; a vervet from Kigali, Rwanda’s densely populated capital, tested positive for *T. p. pertenue*. At least 1 animal of each species tested in Kibale National Park, Uganda, was positive; Kibale contains one of Africa’s most diverse primate communities, and skin lesions consistent with yaws have been documented there for >50 years (*9*) (Figure 1).

**Table 1:** Seroprevalence of *Treponema pallidum pertenue* in nonhuman primates from Uganda and Rwanda*

Species	Country	Location	No. tested	No. positive	Prevalence, % (95% CI)
Black-and-white colobus (*Colobus guereza*)	Uganda	Kibale NP	9	1	11.1 (0.0–45.7)
Eastern chimpanzee (*Pan troglodytes schweinfurthii*)	Uganda	Kibale NP	6	1	16.7 (1.1–58.2)
Golden monkey (*Cercopithecus kandti*)	Rwanda	Volcanoes NP	2	0	0 (0.0–71.0)
L'Hoest’s monkey (*Allocrocebus lhoesti*)	Rwanda	Nyungwe NP	7	0	0 (0.0–40.4)
Mountain gorilla (*Gorilla beringei beringei*)	Rwanda	Volcanoes NP	6	0	0 (0.0–44.3)
Olive baboon (*Papio anubis*)	Both	All locations	26	20	76.9 (57.6–89.3)
	Uganda	Kibale NP	17	11	64.7 (41.2–82.8)
	Rwanda	Akagera NP	9	9	100 (65.5–100)
Red colobus (*Piliocolobus tephrosceles*)	Uganda	Kibale NP	21	2	9.5 (1.5–30.1)
Red-tailed guenon (*Cercopithecus ascanius*)	Uganda	Kibale NP	11	6	54.6 (28.0–78.8)
Vervet (*Chlorocebus pygerythrus*)	Both	All locations	15	4	26.7 (10.5–52.4)
	Uganda	Nabugabo	3	1	33.33 (5.6–79.8)
	Rwanda	Kigali	8	1	12.5 (0.1–49.2)
	Rwanda	Nyungwe NP	2	0	0 (0.00–71.0)
	Rwanda	Akagera NP	2	2	100 (29.0–100)
Total, Uganda	Uganda	All locations	67	22	32.8 (22.8–44.8)
Total, Rwanda	Rwanda	All locations	36	12	33.3 (20.1–49.8)
Total, Uganda and Rwanda	Both	All locations	103	34	33.0 (24.7–42.6)

*NP, national park.

In July 2013 we observed an outbreak of yaws-like disease in a social group of Ugandan red colobus monkeys (*Piliocolobus tephrosceles*) in Kibale. Approximately half the animals displayed skin lesions, including papules, ulcers, and crusts visible on hairless regions (face, plantar surfaces of hands and feet, anogenital region; Figure 1), and ≈10% of animals were not seen again. Animals from the group had been sampled in 2012, before the outbreak, and again in 2014, after the outbreak. Seven animals (33.3%) seroconverted, and 2 seropositive animals from 2012 remained seropositive in 2014 (Table 2). Those data demonstrate active transmission of *T. p. pertenue*, persistence of antibodies for >2.4 years, and an incidence rate during this period of 4.3 (95% CI 1.9 – 9.0) cases/1,000 monkey-months.

**Table 2:** Seroconversion of red colobus monkeys (*Piliocolobus tephrosceles*) to *Treponema pallidum pertenue* in Kibale National Park, Uganda, after an outbreak of yaws, July 2013

Sex	Age at 1st sampling in 2012	Age at 2nd sampling in 2014	Days between samplings	Test result at 1st sampling in 2012	Test result at 2nd sampling in 2014
F	Adult	Adult	1,722	Negative	Positive
F	Adult	Adult	990	Negative	Negative
F	Adult	Adult	995	Negative	Positive
F	Adult	Adult	758	Negative	Positive
F	Adult	Adult	759	Negative	Negative
M	Subadult	Adult	1,073	Negative	Positive
M	Subadult	Adult	940	Negative	Negative
M	Subadult	Adult	823	Negative	Negative
M	Subadult	Adult	796	Negative	Negative
M	Subadult	Adult	726	Negative	Negative
M	Adult	Adult	1,590	Negative	Negative
M	Adult	Adult	612	Negative	Positive
M	Adult	Adult	1,080	Negative	Positive
M	Adult	Adult	934	Negative	Negative
M	Adult	Adult	834	Negative	Negative
M	Adult	Adult	893	Positive	Positive
M	Adult	Adult	835	Positive	Positive
M	Adult	Adult	869	Negative	Negative
M	Adult	Adult	784	Negative	Negative
M	Adult	Adult	794	Negative	Negative
M	Adult	Adult	726	Negative	Positive

We observed 2 more outbreaks in the same red colobus group in January 2015 and December 2017–January 2018; again, ≈50% of animals were affected and ≈10% were not seen again. An adult female red colobus was found moribund on January 12, 2015, and died several hours later. Another adult female was found freshly dead on December 26, 2017. We collected swabs of facial ulcers from both carcasses, placed them in RNAlater (Thermo Fisher Scientific, https://www.thermofisher.com), and stored them at −20°C. Samples tested positive by diagnostic PCR. Hybridization capture yielded bacterial genome sequences of 98% completeness for the 2015 sample and 86% for the 2017 sample (Appendix).

**Figure 2:**
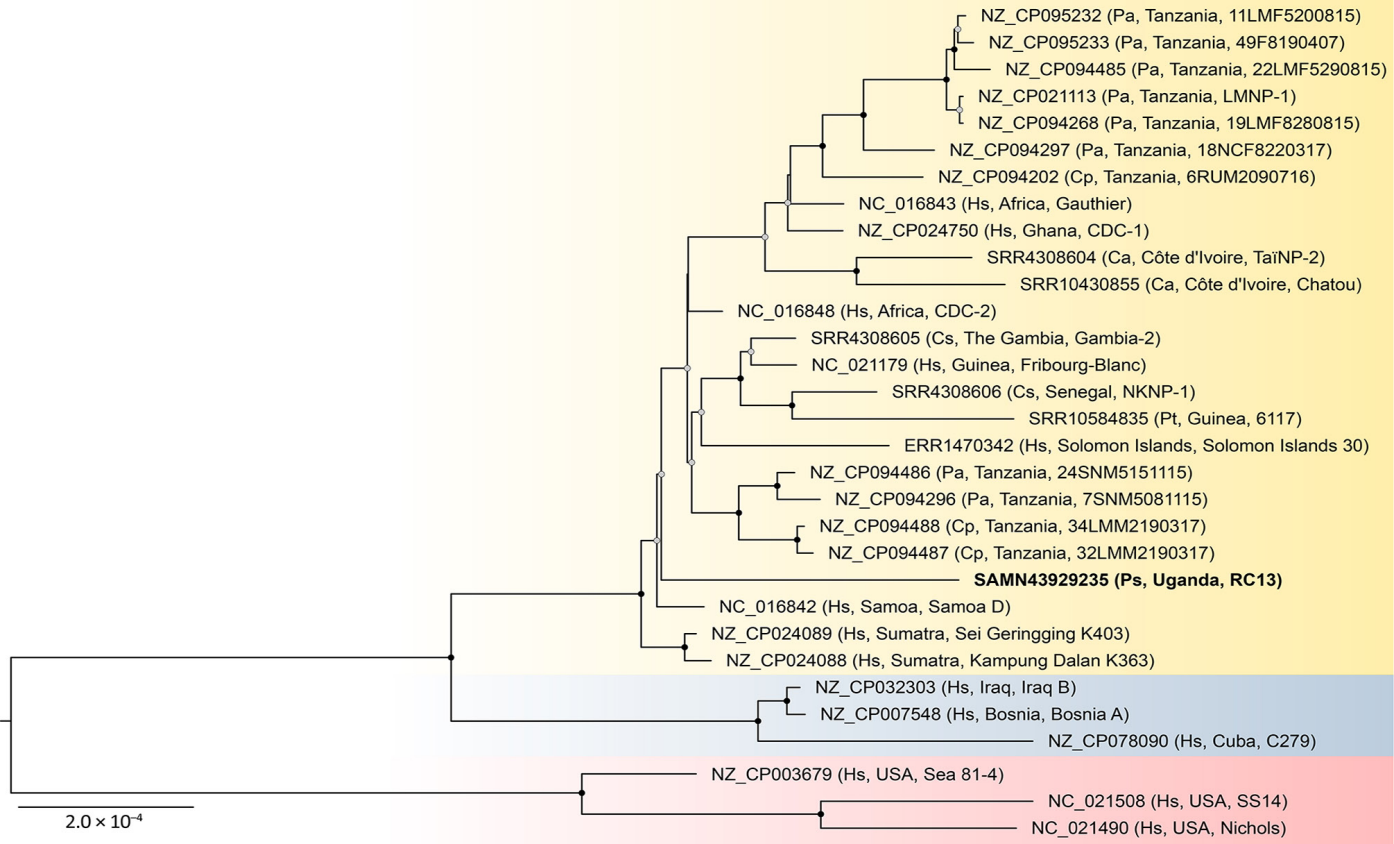
Maximum-likelihood phylogenetic tree of *Treponema pallidum* genomes from study of yaws circulating in nonhuman primates, Uganda and Rwanda. The tree shows relationships among *T. p. pertenue* genomes (yellow) and is rooted with *T. p. endemicum* (blue) and *T. p. pallidum* (red) genomes. Bold text indicates sequence generated in this study from a Ugandan red colobus monkey. Taxon names include GenBank accession numbers, followed in parentheses by primate host, location of origin, and isolate name. The tree was inferred from a 1,072,667-position cleaned nucleotide alignment of 31 nonredundant sequences available in GenBank having genome coverage >97% and containing 4,716 variable positions. Black dots on nodes indicate bootstrap values of 100%; gray dots indicate 75%–99% bootstrap values based on 1,000 bootstrap replicates; values <75% are not shown. Scale bar indicates nucleotide substitutions per site. Ca, *Cercocebus atys*; Cp, *Chlorocebus pygerythrus*; Cs, *Cercocebus sabaeus*; Hs, *Homo sapiens*; Pa, *Papio anubis*; Ps, *Piliocolobus tephrosceles*; Pt, *Pan troglodytes.*

A phylogenetic tree of reference sequences with >97% genome coverage showed the variant in the 2015 Ugandan red colobus to be a distinct lineage (Figure 2). Another phylogenetic tree (Appendix Figure 2) including the 86% complete 2017 outbreak sequence and a 57% complete Western red colobus (*Procolobus badius*) sequence showed the 2 Ugandan red colobus variants to be sister taxa but distinct from the Western red colobus variant. Despite the 2015 and 2017 outbreaks occurring in the same social group, the 2 outbreak sequences differed at 2,131/976,212 (0.2%) nucleotide positions (omitting gaps), demonstrating circulation of multiple *T. p. pertenue* variants among primates in the area.

Infection of primates with *T. p. pertenue* was widespread in Uganda and Rwanda at the time of sampling, similar to other locations in sub-Saharan Africa (*5*,*6*). Overall, one third of primates tested had antibodies to *T. p. pertenue*, comparable to 53% seroprevalence found in Tanzania primates (*10*). Seropositivity in Uganda and Rwanda increased with age and varied widely by species and location. Olive baboons had the highest rate of seropositivity (77%), followed by red-tailed guenons (55%) and vervet monkeys (27%). Olive baboons and vervet monkeys live alongside humans throughout their range, which could enable zoonotic transmission. All species tested from Kibale had >1 positive animal, which is consistent with historical reports of yaws-like disease in Kibale primates (*9*). Those species include the eastern chimpanzee (*Pan troglodytes schweinfurthii*); because western chimpanzees (*P. t. verus*) are also infected with *T. p. pertenue* (*11*), *T. p. pertenue* likely infects chimpanzees across their range. Although no L’Hoest’s monkeys (*Allochrocebus lhoesti*) from Rwanda were seropositive, L’Hoest’s monkeys in Kibale frequently display yaws-like lesions (Figure 1), again demonstrating wide geographic variation in infection.

## Conclusions

Our results provide direct evidence of active *T. p. pertenue* circulation in Ugandan red colobus, in which yaws-like disease has been documented for >50 years (*9*). After an outbreak, 33% of red colobus in a social group seroconverted. Of interest, the 2015 and 2017 outbreak strains from this social group differed genetically. Although the rate of *T. p. pertenue* evolution in primates remains unknown, it is unlikely that the 2017 variant was a direct descendent of the 2015 variant, given >2,000 nt differences. We suspect that a diversity of *T. p. pertenue* variants circulates in and among primate species in the area. Moreover, the newly sequenced Ugandan red colobus variants were distinct from previously published sequences, including a variant from a West African red colobus monkey. Our phylogenetic analyses support the idea that *T. p. pertenue* evolution has been shaped by geography, reflected by subclades tending to consist of variants from similar locations, including interspersion of human and primate variants, as previous studies have also found (*7*,*8*). If so, *T. p. pertenue* may be maintained in primates through localized cycles of transmission, limited more by geographic distance than by host species.

Facial deformities of Kibale primates have been variously attributed to congenital malformation and agricultural chemicals (*12*,*13*). Our results, combined with growing evidence from across sub-Saharan Africa, strongly suggest that the actual cause is yaws. Ugandan red colobus monkeys are endangered, existing mostly in small, geographically isolated populations (*9*). The effects of lethal yaws outbreaks could be significant for these and other primates, especially for populations simultaneously facing habitat loss, fragmentation, hunting, and other anthropogenic stressors (*14*).

Uganda and Rwanda are considered previously endemic countries for yaws but not endemic as of March 2025 (*3*). The World Health Organization has targeted yaws eradication by 2030, but barriers remain, and primate reservoirs would add substantially to these barriers (*3*,*4*). Yaws outbreaks have not been reported recently in humans living near the primates tested, despite frequent close interaction (including direct contact). Examining host specificity of *T. p. pertenue* variants and epidemiologic barriers to zoonotic transmission would help elucidate whether primates could seed new human infections in currently endemic countries or reintroduce the disease to humans in previously endemic countries.

Appendix Additional information about yaws circulating in nonhuman primates, Uganda and Rwanda.
