# Supplementary material for: Yaws Circulating in Nonhuman Primates, Uganda and Rwanda
Source: Emerg Infect Dis. 2025 Apr;31(4):799–803. doi: 10.3201/eid3104.241562 (PMC11950284; doi:10.3201/eid3104.241562)
Supplement: Appendix — Additional information about yaws circulating in nonhuman primates, Uganda and Rwanda. [file 24-1562-Techapp-s1.pdf]

Article DOI: https://doi.org/10.3201/eid3104.241562

EID cannot ensure accessibility for supplementary materials supplied by authors. Readers who have difficulty accessing supplementary content should contact the authors for assistance.

Yaws Circulating in Nonhuman Primates, Uganda and Rwanda

Appendix

Samples and Serology

Primate serum samples from existing collections were used for this study. Sera were originally collected from six locations in Uganda and Rwanda from 2005–2017 (Appendix Figure 1) as part of multiple efforts to characterize infectious diseases in primates from these countries (*1*,*2*). Primates were anesthetized in the field, sampled, and released to their social groups as previously described (*3*,*4*). Serum was separated from whole blood by centrifugation and immediately frozen in liquid nitrogen. Samples were stored in liquid nitrogen or in −80°C freezers and were shipped in liquid nitrogen dry shippers to the USA for analysis (see ethics statement in main article).

We used a commercial passive particle agglutination test (SERODIA-TP•*PA*; Fujirebio Diagnostics, Tokyo, Japan) to detect *T. pallidum* antibodies. This test is 97.7% sensitive and 92.0% specific for antibodies to *T. pallidum* in nonhuman primates and has the advantage that it can be read visually and can be performed in resource poor laboratories (*5*). Although the test detects antibodies to *T. pallidum* in general and does not distinguish among bacterial subspecies, positivity in wild nonhuman primates can be interpreted as indicating infection with *T. p. pertenue* because wild primates are not known to harbor *T. p. pallidum* (the cause of syphilis) or *T. p. endemicum* (the cause of bejel) but are commonly infected with *T. p. pertenue* (*6*). We performed tests in duplicate using 25 µl of serum following the manufacturer's protocol, including negative and positive control standards.

PCR Testing

We tested swab samples using endpoint PCR targeting the TPE *gdp* gene (*7*,*8*). We extracted DNA from swab tips using the DNeasy Blood & Tissue Kit (Qiagen, Hilden, Germany) and eluted it into 100 µl of kit-provided buffer, then we conducted PCR amplifications in 25µl volumes using the HotStarTaq kit (Qiagen) according to the manufacturer's instructions, with primers gdp-f (5′-AAGAACTTTCCCTCCTCCGTGC-3′) and gdp-r (5′-AAGAACTTTCCCTCCTCCGTGC-3′), 2 µl DNA extract as template, and the following thermal profile: 95°C for 15m; 35 cycles of 94°C for 30s, 57°C for 30s, 72°C for 30s; and 72°C for 10m. We electrophoresed PCR products on 1% agarose gels stained with ethidium bromide, visualized them under ultraviolet light, excised amplicons using sterile razor blades, and extracted them from the agarose matrix using the Zymoclean Gel DNA Recovery Kit (Zymo Research, Irvine, CA, USA). To confirm amplification of TPE *gdp*, we performed Sanger sequencing on ABI 3730xl sequencers (Thermo Fisher Scientific, Waltham, MA, USA) at a commercial facility (Functional Biosciences, Madison, WI, USA).

Library Preparation, Genome-Wide Capture, and High-Throughput Sequencing

We converted DNA into dual-indexed libraries using the NEBNext Ultra II FS DNA Library Prep Kit for Illumina (New England Biolabs, Ipswich, MA, USA) with a 12-minute incubation at 37°C for fragmentation and with adaptors from the NEBNext Multiplex Oligos for Illumina kit (New England Biolabs). We quantified/analyzed all generated libraries with a Qubit fluorometer using a double-stranded DNA high-sensitivity assay kit (Thermo Fisher Scientific, Waltham, MA, USA) and an Agilent BioAnalyzer (Agilent Technologies, Santa Clara, CA, USA), following the instructions of the manufacturers. We enriched libraries for *T. pallidum* sequences using in-solution hybridization capture with biotinylated RNA baits (myBaits, Arbor Biosciences, Ann Arbor, MI, USA) spanning the simian-derived Fribourg-Blanc reference genome (RefSeq ID: NC_021179.1) with 2-fold tiling as previously described (*9*). Briefly, we performed 2 × 48 hour in-solution hybridization captures and, after each round of capture. we performed a KAPA HiFi HotStart library amplification (KAPA Biosystems, Wilmington, MA, USA) with P5 and P7 primers (Illumina, San Diego, CA, USA) to generate ~200 ng of enriched DNA per sample, using the thermal profile: 98 °C for 2m;12 to 16 cycles of 98 °C for 20s; 65 °C for 30 s; 72 °C for 45s; and 72 °C for 5m. We quantified/analyzed enriched libraries with a Qubit

fluorometer (Thermo Fisher Scientific) and an Agilent BioAnalyzer following the instructions of the manufacturers. Prior to sequencing, we diluted libraries to 4 nM and pooled them for sequencing on an Illumina MiSeq instrument with 300 bp paired-end read V3 chemistry.

Bioinformatics

We analyzed sequence data using CLC Genomics Workbench version 23.0.2 (Qiagen). We trimmed low-quality bases (Phred quality score <30), discarded short reads (<30 bp), merged paired reads, and mapped merged and singleton reads to the TPE Fribourg-Blank reference sequence (RefSeq ID NC_021179.1) using length fraction 0.7 and similarity fraction 0.8. We then merged mapped reads, removed duplicate reads, and called a consensus genome using a minimum coverage of 3 unique reads, applying a majority consensus rule, and filling in "N" for areas of low coverage (<3 unique reads).

Phylogenetics

We aligned newly generated nucleotide sequences with published sequences using MAFFT (*10*) and removed poorly aligned regions using trimAl (*11*). We removed recombinant genes, genes under positive selection, and hypervariable genes containing repetitive regions to improve the accuracy of phylogenetic inference (*12–15*). We then inferred phylogenetic trees using IQ-TREE (*16*) with a substitution model estimated from the data and 1,000 ultrafast bootstrap replicates to assess statistical support for clades. We displayed resulting phylogenetic trees using FigTree version 1.4.4 (*17*).

References

1. Goldberg TL, Paige SB, Chapman CA. The Kibale EcoHealth Project: exploring connections among human health, animal health, and landscape dynamics in western Uganda. In: Aguirre AA, Daszak P, Ostfeld RS, editors. New directions in conservation medicine: applied cases of ecological health. New York: Oxford University Press; 2012. p. 452–65.

2. Nyatanyi T, Wilkes M, McDermott H, Nzietchueng S, Gafarasi I, Mudakikwa A, et al. Implementing One Health as an integrated approach to health in Rwanda. BMJ Glob Health. 2017;2:e000121. PubMed https://doi.org/10.1136/bmjgh-2016-000121

3. Lauck M, Hyeroba D, Tumukunde A, Weny G, Lank SM, Chapman CA, et al. Novel, divergent simian hemorrhagic fever viruses in a wild Ugandan red colobus monkey discovered using direct pyrosequencing. PLoS One. 2011;6:e19056. PubMed https://doi.org/10.1371/journal.pone.0019056

4. Hyeroba D, Apell P, Goldberg T, Shafer LA, Kidega T, Asimwe C. Ketamine-medetomidine regimen for chemical immobilisation of free-ranging chimpanzees (*Pan troglodytes schweinfurthii*) in Uganda. Vet Rec. 2013;172:475. 36 PubMed https://doi.org/10.1136/vr.101336

5. Knauf S, Dahlmann F, Batamuzi EK, Frischmann S, Liu H. Validation of serological tests for the detection of antibodies against *Treponema pallidum* in nonhuman primates. PLoS Negl Trop Dis. 2015;9:e0003637. PubMed https://doi.org/10.1371/journal.pntd.0003637

6. Chuma IS, Abel L, Hallmaier–Wacker LK, Šmajs D, Knauf S. Pathogenic spirochetes in monkeys: stealthy pathogens of global importance. In: Knauf S, Jones-Engel L, editors. Neglected diseases in monkeys: from the monkey-human interface to One Health. Cham, Switzerland: Springer; 2020. p. 95–119.

7. Harper KN, Ocampo PS, Steiner BM, George RW, Silverman MS, Bolotin S, et al. On the origin of the treponematoses: a phylogenetic approach. PLoS Negl Trop Dis. 2008;15;2:e148.

8. Gogarten JF, Düx A, Schuenemann VJ, Nowak K, Boesch C, Wittig RM, et al. Tools for opening new chapters in the book of *Treponema pallidum* evolutionary history. Clin Microbiol Infect. 2016;22:916–21. PubMed https://doi.org/10.1016/j.cmi.2016.07.027

9. Mubemba B, Gogarten JF, Schuenemann VJ, Düx A, Lang A, Nowak K, et al. Geographically structured genomic diversity of non-human primate-infecting *Treponema pallidum* subsp. *pertenue.* Microb Genom. 2020;6:mgen000463. PubMed https://doi.org/10.1099/mgen.0.000463

10. Katoh K, Misawa K, Kuma K, Miyata T. MAFFT: a novel method for rapid multiple sequence alignment based on fast Fourier transform. Nucleic Acids Res. 2002;15:30:3059–66.

11. Capella-Gutierrez S, Silla-Martinez JM, Gabaldon T. trimAl: a tool for automated alignment trimming in large-scale phylogenetic analyses. Bioinformatics. 2009;25:1972–3.

12. Maděránková D, Mikalová L, Strouhal M, Vadják Š, Kuklová I, Pospíšilová P, et al. Identification of positively selected genes in human pathogenic treponemes: syphilis-, yaws-, and bejel-causing strains differ in sets of genes showing adaptive evolution. PLoS Negl Trop Dis. 2019;13:e0007463. PubMed https://doi.org/10.1371/journal.pntd.0007463

13. Mubemba B, Chanove E, Mätz-Rensing K, Gogarten JF, Düx A, Merkel K, et al. Yaws disease caused by *Treponema pallidum* subspecies *pertenue* in wild chimpanzee, Guinea, 2019. Emerg Infect Dis. 2020;26:1283–6. PubMed https://doi.org/10.3201/eid2606.191713

14. Janečková K, Roos C, Fedrová P, Tom N, Čejková D, Lueert S, et al. The genomes of the yaws bacterium, *Treponema pallidum* subsp. *pertenue*, of nonhuman primate and human origin are not genomically distinct. PLoS Negl Trop Dis. 2023;17:e0011602. PubMed https://doi.org/10.1371/journal.pntd.0011602

15. Majander K, Pla-Díaz M, du Plessis L, Arora N, Filippini J, Pezo-Lanfranco L, et al. Redefining the treponemal history through pre-Columbian genomes from Brazil. Nature. 2024;627:182–8. PubMed https://doi.org/10.1038/s41586-023-06965-x

16. Nguyen L-T, Schmidt HA, von Haeseler A, Minh BQ. IQ-TREE: a fast and effective stochastic algorithm for estimating maximum-likelihood phylogenies. Mol Biol Evol. 2015;32:268–74. PubMed https://doi.org/10.1093/molbev/msu300

17. Rambaut A. FigTree, v1.4.4. 2018 [cited 2024 Oct 14]. https://github.com/rambaut/figtree/releases/tag/v1.4.4.

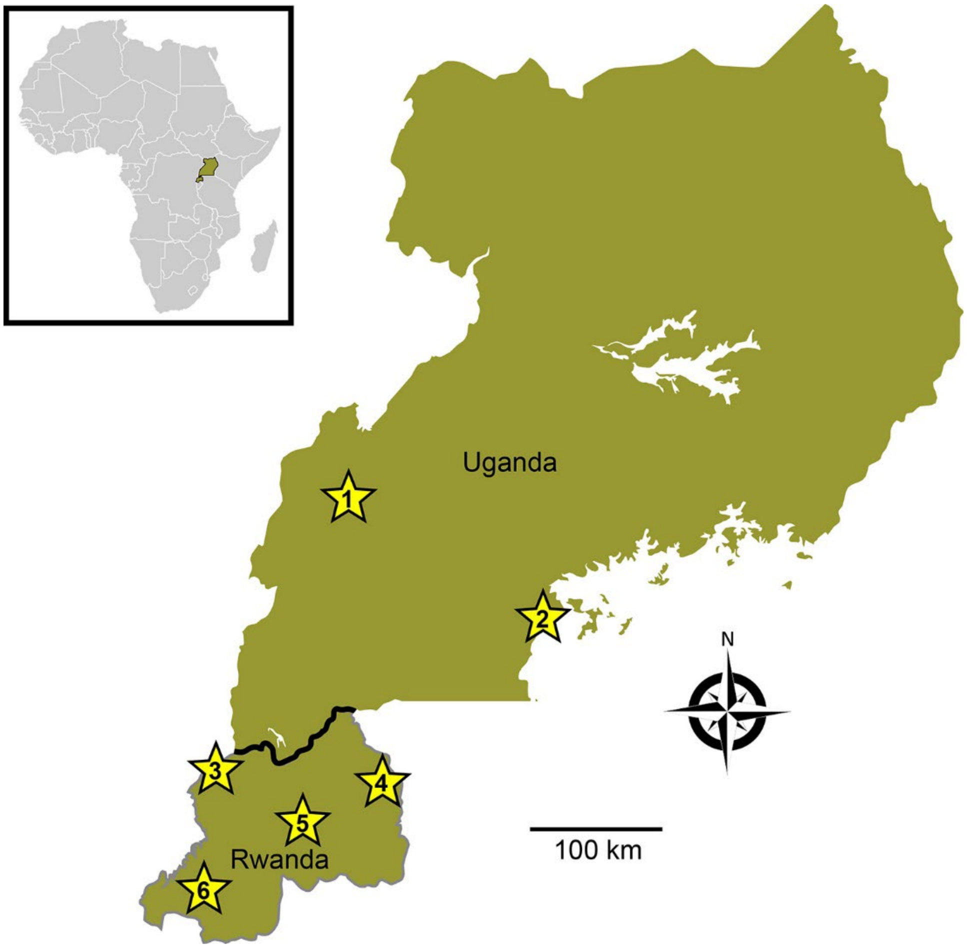

Appendix Figure 1. Map of sampling locations. Insert shows Uganda and Rwanda within Africa. Yellow stars indicate sampling locations within Uganda and Rwanda: 1 = Kibale National Park; 2 = Lake Nabugabo; 3 = Volcanoes National Park; 4 = Akagera National Park; 5 = Kigali; 6 = Nyungwe National Park.

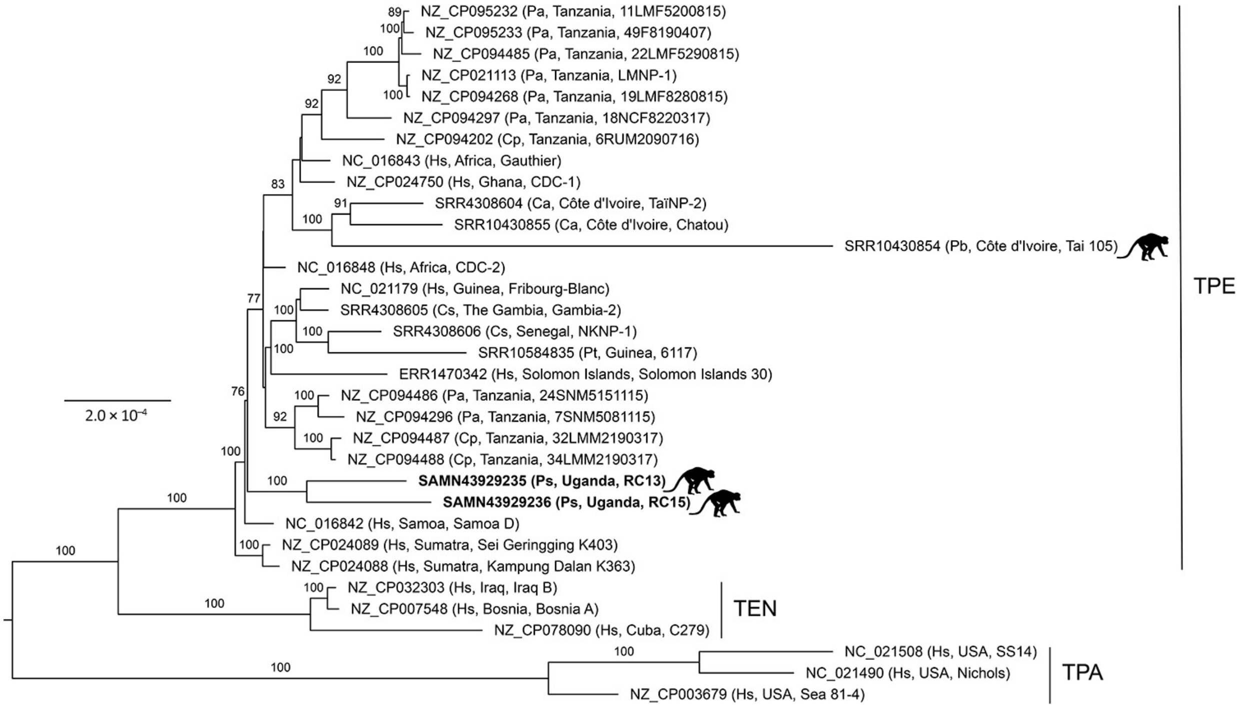

Appendix Figure 2. Maximum likelihood phylogenetic tree of *Treponema pallidum* genomes. The tree shows relationships among *T. p. pertenue* genomes (TPE) and is rooted with *T. p. endemicum* (TEN) and *T. p. pallidum* (TPA) genomes. The tree contains the same taxa as Figure 2, except for the addition of RC13 (86% complete, from a Ugandan red colobus in 2017) and SRR10430854 (57% complete, from a West African red colobus in 2013). Taxon names include GenBank accession numbers followed in parentheses by primate host (Ca = *Cercocebus atys*; Cp = *Chlorocebus pygerythrus*; Cs = *Cercocebus sabaeus*; Hs = *Homo sapiens*; Pa = *Papio anubis*; Pb = *Procolobus badius*; Ps = *Piliocolobus tephrosceles*; Pt = *Pan troglodytes*), location of origin, and isolate name. The tree was inferred from a 1,072,401-position cleaned nucleotide alignment containing 3,930 variable positions. Silhouettes indicate sequences from red colobus; sequences generated in this study are in bold. Numbers indicate bootstrap values based on 1,000 replicates (values <75% are not shown). Scale bar indicates nucleotide substitutions per site.
